# Risk Factors of Secondary Cardiovascular Events in a Multi-Ethnic Asian Population with Acute Myocardial Infarction: A Retrospective Cohort Study from Malaysia

**DOI:** 10.3390/jcdd10060250

**Published:** 2023-06-09

**Authors:** Sophia Rasheeqa Ismail, Mohd Shawal Faizal Mohammad, Adam S. Butterworth, Rajiv Chowdhury, John Danesh, Emanuele Di Angelantonio, Simon J. Griffin, Lisa Pennells, Angela M. Wood, Mohd Fairulnizal Md Noh, Shamsul Azhar Shah

**Affiliations:** 1 Nutrition, Metabolic and Cardiovascular Research Centre, Institute for Medical Research, National Institutes of Health, Ministry of Health Malaysia, Shah Alam 40170, Malaysia; sophia.rasheeqa@moh.gov.my (S.R.I.);; 2 Department of Community Health, Faculty of Medicine, National University of Malaysia, Kuala Lumpur 56000, Malaysia; 3 Department of Cardiology, Hospital Canselor Tuanku Muhriz, Kuala Lumpur 56000, Malaysia; 4 British Heart Foundation Cardiovascular Epidemiology Unit, Department of Public Health and Primary Care, University of Cambridge, Cambridge CB2 0BB, UKed303@medschl.cam.ac.uk (E.D.A.);; 5 British Heart Foundation Centre of Research Excellence, University of Cambridge, Cambridge CB2 0BB, UK; 6 National Institute for Health and Care Research Blood and Transplant Research Unit in Donor Health and Behaviour, University of Cambridge, Cambridge CB2 0BB, UK; 7 Health Data Research UK Cambridge, Wellcome Genome Campus, University of Cambridge, Cambridge CB10 1SA, UK; 8 Stempel College of Public Health and Social Work, Florida International University, Miami, FL 33174, USA; 9 Department of Human Genetics, Wellcome Sanger Institute, Cambridge CB10 1SA, UK; 10 Health Data Science Research Centre, Human Technopole, 20157 Milan, Italy; 11 Department of Public Health and Primary Care, University of Cambridge, Cambridge CB2 0BB, UK; 12 MRC Epidemiology Unit, School of Clinical Medicine, University of Cambridge, Cambridge CB2 0SL, UK; 13 The Alan Turing Institute, London NW1 2DB, UK; 14 Medical Research Council Biostatistics Unit, Cambridge Institute of Public Health, University of Cambridge, Cambridge CB2 0SR, UK

**Keywords:** myocardial infarction, risk factors, major adverse cardiovascular events, cardiovascular mortality, Asian

## Abstract

This retrospective cohort study investigated the incidence and risk factors of major adverse cardiovascular events (MACE) after 1 year of first-documented myocardial infarctions (MIs) in a multi-ethnic Asian population. Secondary MACE were observed in 231 (14.3%) individuals, including 92 (5.7%) cardiovascular-related deaths. Both histories of hypertension and diabetes were associated with secondary MACE after adjustment for age, sex, and ethnicity (HR 1.60 [95%CI 1.22–2.12] and 1.46 [95%CI 1.09–1.97], respectively). With further adjustments for traditional risk factors, individuals with conduction disturbances demonstrated higher risks of MACE: new left-bundle branch block (HR 2.86 [95%CI 1.15–6.55]), right-bundle branch block (HR 2.09 [95%CI 1.02–4.29]), and second-degree heart block (HR 2.45 [95%CI 0.59–10.16]). These associations were broadly similar across different age, sex, and ethnicity groups, although somewhat greater for history of hypertension and BMI among women versus men, for HbA1c control in individuals aged >50 years, and for LVEF ≤ 40% in those with Indian versus Chinese or Bumiputera ethnicities. Several traditional and cardiac risk factors are associated with a higher risk of secondary major adverse cardiovascular events. In addition to hypertension and diabetes, the identification of conduction disturbances in individuals with first-onset MI may be useful for the risk stratification of high-risk individuals.

## 1. Introduction

Individuals who have had a recent acute myocardial infarction (MI) event are at a higher risk of secondary major adverse cardiovascular events (MACE) [1]. These individuals have at least 30% higher morbidity and mortality risks than the general population [2]. The outcomes of an atherosclerotic acute MI event are generally influenced by the patient’s premorbid risk factors, the extent of infarction, and arrhythmias, as well as the consequent mechanical dysfunctions. Although there is considerable overlap between the risk factors of the first MI event and MACE, variabilities in risk factors for secondary MACE have been reported in different populations [3,4,5,6]. Thus, the identification of risk factors of MACE is particularly important in multi-ethnic populations for the appropriate planning of treatment strategies.

Malaysia is home to 60 native ethnic groups such as Malays and Orang Asli from the Malay Peninsula, Kadazandusun, and Iban from the Malay parts of Borneo. Collectively, these native ethnic groups are referred to as Bumiputera, and represent the largest proportion of the Malaysian population followed by Chinese and Indians. The prevalence of traditional risk factors for coronary heart disease (CHD) is prominent in this multi-ethnic population [7,8]. However, its cultural and genetic diversity may also contribute to the distinct cardiovascular disease morbidity and mortality risks that have been observed compared to Western populations [9,10,11].

The distinct population and cardiovascular risk profiles warrant further evaluation of the secondary MACE in this population. This study aimed to complement the findings generated by the Malaysian National Cardiovascular Disease–Acute Coronary Syndrome (NCVD–ACS) Registry in understanding local disease patterns that could inform the provision of local resources for intervention. Therefore, we specifically aimed at investigating the incidence of secondary MACE and their related risk factors in individuals with the first documented MI in Malaysia. Additionally, we evaluated the differences in risk factors for secondary MACE across age, gender, and ethnic groups.

## 2. Materials and Methods

This cohort study retrospectively followed up on MI cases recruited in the MAVERIK study. The baseline information was retrieved from the MAVERIK study database and hospital admission records, whereas the outcomes data were retrieved from the hospital admission records and the National Registration Department. The data were collected by trained study coordinators who used an electronic standardized questionnaire. No active enrolment nor laboratory analyses were conducted in this study.

This study was approved by the Malaysian Medical Research and Ethics Committee (Ref: KKM/NIHSEC/P19-2561(10)). The research was conducted in accordance with the Declaration of Helsinki. All the participants provided written informed consent prior to their participation in the MAVERIK study. All the analyses were performed with anonymized data.

### 2.1. Malaysian Acute Vascular Events Risk (MAVERIK) Study and the Study Population

We studied individuals with a first-documented MI event recruited from the MAVERIK study. The MAVERIK study is a multi-center hospital-based case-control study of genetic, lifestyle, and other determinants of acute MI in Malaysia, described previously [12]. Briefly, the MAVERIK study was conducted between June 2017 and June 2019. Serum and whole blood samples were collected and stored. Additionally, clinical, demographic, and an 83-item questionnaire were collected for each participant.

The MI cases were recruited if they were Malaysians residing in Malaysia, male or female aged 18 years old and above, who were present at the hospital after the onset of sustained clinical symptoms that were suggestive of MI and lasting longer than 20 min, and had a diagnosis of MI, either ST-segment elevation myocardial infarction (STEMI) or non-ST segment elevation myocardial infarction (NSTEMI), based on the Malaysia Clinical Practice Guidelines [13,14]. Moreover, the cases had no previous documented cardiovascular disease events (defined as previous MI, unstable angina, heart failure, cardiac arrhythmia, rheumatic heart disease, infective endocarditis, transient ischemic attack, stroke, peripheral vascular disease, and other CVD or evidence of coronary heart disease on prior electrocardiograms or in medical hospital records) and were not concurrently hospitalized for any other CVD events.

### 2.2. MACE Outcome

The primary outcome was the first incidence of a secondary non-fatal or fatal MACE within 1 year after the index MI.

Non-fatal MACE were defined as composite non-fatal cardiovascular events that included hospital admission for acute coronary syndrome (ACS)(including recurrent NSTEMI, recurrent STEMI, and unstable angina), stable angina, admission for heart failure (including symptomatic heart failure, congestive heart failure, decompensated congestive heart failure), stroke (including ischemic stroke, hemorrhagic stroke, or transient ischemic attack), and revascularization (including repeated percutaneous coronary intervention [PCI] or coronary artery bypass graft after the initial intervention).

Fatal MACE were defined as any cardiovascular-related deaths, and included International Classification of Diseases (ICD)-10 codes I10–I15 (hypertensive diseases), I20–25 (ischemic heart diseases), I60–I69 (cerebrovascular diseases), and I70 and I71 (other atherosclerosis). The mortality statuses were retrieved from the Malaysian National Registration Department in November 2020.

### 2.3. Baseline Characteristics and Exposures

The baseline characteristics and exposures of interest were obtained from the MAVERIK study database and hospital medical records, and encompassed the following: (1) demographics including age, sex, and self-reported ethnicity (classified as Bumiputera, Chinese, and Indian); (2) comorbidities including self-reported histories of hypertension, diabetes, dyslipidemia, current smoking status (versus no/former smoking status), and body mass index (BMI); (3) blood parameters from non-fasting blood samples collected by the MAVERIK study including total cholesterol (TC), low-density lipoprotein (LDL), high-density lipoprotein (HDL), triglyceride, lipoprotein (a) and HbA1c; and (4) cardiac characteristics including the type of baseline MI, arrhythmic complications (new onset of left-bundle branch block [LBBB], right-bundle branch block [RBBB], heart blocks, atrial fibrillation, ventricular fibrillation, and supraventricular tachycardia) and mechanical complications (documented presence of heart failure, left ventricular ejection fraction [classified as ≤40%, 41–49%, and ≥50% according to the ESC Guidelines [15], cardiogenic shock, and cardiac arrest). Evidence-based cut-off values were applied for several biomarkers to differentiate between higher and lower values: HbA1c > 6.5%, LDL > 2.6 mmol/L [16], lipoprotein (a) >30 mg/dL [17], and BMI ≥ 25 kg/m^2^ [18].

### 2.4. Statistical Analysis

Hazard ratios (HRs) were used to quantify the associations between exposures and secondary MACE. The analyses used Cox proportional hazards models with the time since the index MI as the timescale. An index MI event was defined as the date of the first documented MI. The censoring was considered to be the earliest of the date of fatal or non-fatal MACE, death from other causes, or 1 year after the index MI. We estimated crude HRs as well as sequentially progressive adjusted HRs, including for age, sex, ethnicity, comorbidities, blood parameters, and cardiac characteristics. The proportional hazards assumption was checked using Schoenfeld residuals and revealed that there was no violation.

Furthermore, we investigated effect modification by age (age ≤50 years vs. >50 years), sex (female vs. male), and ethnicity (Bumiputera vs. Chinese vs. Indian) groups by including the relevant interaction terms. The confidence intervals were set at 95%, with a *p*-value of less than 0.05 as being statistically significant. The analyses were performed in R software (version 4.0.1) (R Core Team 2020).

## 3. Results

There were 1,618 individuals in the MAVERIK study who were identified with a first-documented MI event and followed-up for up to 1 year. The cohort participants had a mean age of 50.1 years old (SD 9.5), of which 786 (47.5%) were aged ≤50 years and 1476 (91.2%) were males. Bumiputera participants represented 61.4% of the cohort.

The known comorbidities, such as hypertension, diabetes, and dyslipidemia, were present in 35.8%, 28.9%, and 23.6% of the cases, respectively. The majority of the cohort participants were active smokers (61.4%) with a BMI ≥ 25 kg/m^2^ (78.6%). In terms of blood parameters, elevated LDL levels were present in 87.1% of the participants, elevated lipoprotein (a) in 41.4%, and elevated HbA1c in 39.9%. Overall, 60.5% of the individuals had undergone a PCI. A PCI was performed in 64.3% of the STEMI individuals and in 53.6% of the NSTEMI individuals. The baseline characteristics of the cohort participants are described in Table 1. The baseline characteristics by age and ethnic groups are summarized in Tables S2 and S3, respectively.

In the individuals with a first-documented MI, the incidence of cardiovascular-related mortality was lower than non-fatal secondary events by the end of the 1-year follow-up period. There were 231 (14.3%) individuals with a secondary MACE, including 113 (7.0%) ACS events, 92 (5.7%) cardiovascular-related deaths, 14 (0.9%) heart failure admissions, 8 (0.5%) stable angina, 3 (0.2%) stroke events, and 1 (0.06%) revascularization event.

### 3.1. Risk Factors Associated with MACE

The female sex was associated with a higher risk of secondary MACE (HR 1.51 [95%CI 1.01–2.24]). However, this significance was lost after adjustments for age and ethnicity (adjusted HR 1.47 [95%CI 0.98–2.18]) (Table S4).

**Figure 1:**
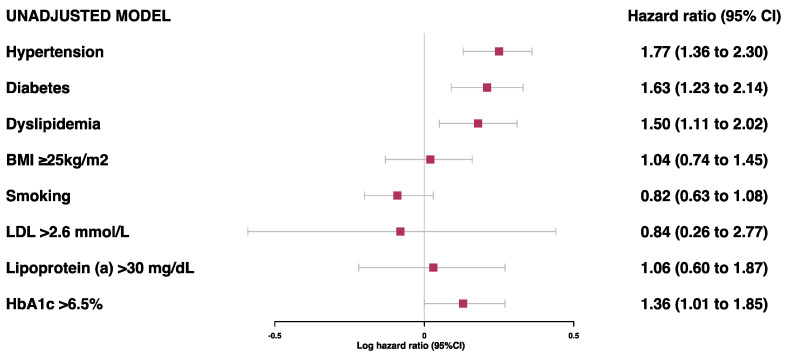
Unadjusted model for associations between traditional risk factors of MI and risk of MACE.

**Figure 2:**
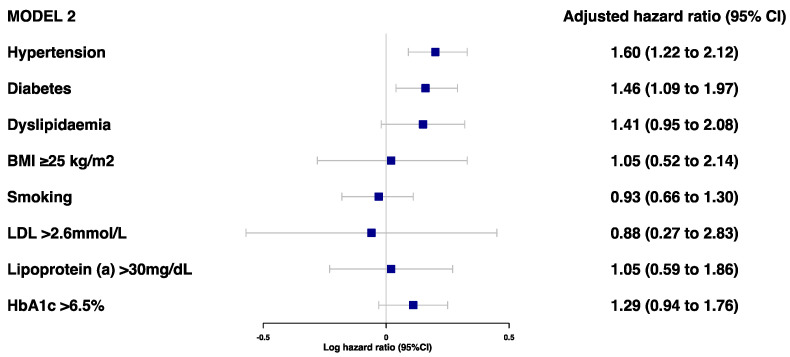
Adjusted model 2 for associations between traditional risk factors of MI and risk of MACE (model 2 was adjusted for age, sex, and ethnicity).

Prior to the adjustments, hypertension, diabetes, dyslipidemia, and HbA1c > 6.5% were found to be associated with a risk of secondary MACE (Figure 1). However, after adjustments for age, sex, and ethnicity, only hypertension (HR 1.60 [95%CI 1.22–2.12]) and diabetes (HR 1.46 [95%CI 1.09–1.97]) were associated with a high risk of MACE (Figure 2 and Table S4). There was no evidence of an association between the risk of secondary MACE with an overweight/obese BMI and current smoking status.

**Figure 3:**
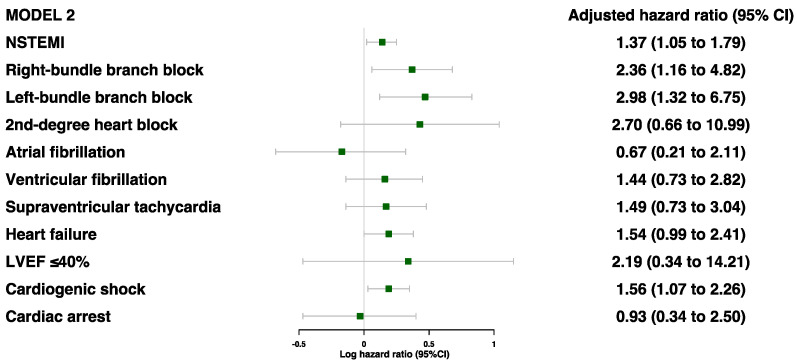
Adjusted model 2 for associations between cardiac characteristics during MI event and risk of MACE (model 2 was adjusted for age, sex, and ethnicity).

**Figure 4:**
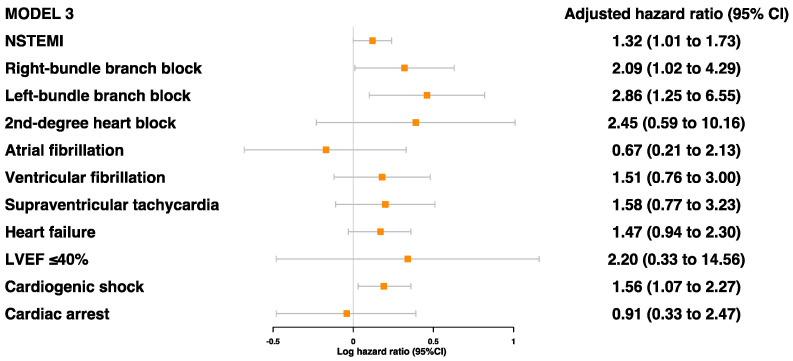
Adjusted model 3 for associations between cardiac characteristics during MI event and risk of MACE (model 3 was adjusted for age, sex, ethnicity, self-reported hypertension, self-reported diabetes, self-reported dyslipidemia, smoking status, BMI, HbA1c, and lipoprotein (a)).

Of the cardiac characteristics, five were found to be significantly associated with secondary MACE prior to adjustments: NSTEMI, RBBB, LBBB, heart failure, and cardiogenic shock (Table S4). After adjustments for age, sex, and ethnicity, all but heart failure was associated with a higher risk of secondary MACE: NSTEMI (HR 1.37 [95%CI 1.05–1.79]), RBBB (HR 2.36 [95%CI 1.16–4.82]), LBBB (HR 2.98 [95%CI 1.32–6.75]), and cardiogenic shock (HR 1.56 [95%CI 1.07–2.27]) (Figure 3 and Table S4). Even after further adjustments for age, sex, ethnicity, comorbidities, and blood parameters, these four risk factors were still found to be significantly associated with secondary MACE (Figure 4).

General patterns of association across all levels of adjustment were seen for several factors, which may warrant further investigation. For example, the presence of second-degree heart block, heart failure, and LVEF ≤ 40% showed HRs of 2.09 (95%CI 0.48–9.14), 1.18 (95%CI 0.69–2.01), and 2.24 (95%CI 0.27–18.39), respectively, after adjustments for age, sex, ethnicity, comorbidities, blood parameters, and other cardiac characteristics (Table S4).

### 3.2. Risk Factors Associated with MACE by Demographics

Of the 768 individuals aged ≤50 years, 101 (13.2%) had secondary MACE, compared to 130 (15.3%) of the 850 individuals aged >50 years (Supplementary File). After adjusting for gender and ethnicity, while the power to detect differences in risk associations across age groups was limited, the association for HbA1c with secondary MACE was potentially greater at older ages: age ≤ 50 years (HR 0.81 [95%CI 0.53–1.24]), age > 50 years (HR 1.57 [95%CI 1.03–2.38]) (*p* = 0.041, testing the null hypothesis of no difference across age groups, Table S5). A potentially important effect modification by age was observed for the presence of second-degree heart block with an HR of 18.27 (95%CI 2.51–133.07) in individuals aged ≤50 years versus 0.08 (95%CI 0.01–1.33) in those aged >50 years on adjustment for age, sex, ethnicity, comorbidities, lipoprotein (a), and HbA1c (Table S5).

There were 28 (19.7%) MACE in female individuals and 203 (13.8%) MACE in male individuals (Table 1). After adjusting for age and ethnicity, the association of diabetes and a high BMI with secondary MACE appeared to be higher for females (HRs 1.72 [95%CI 1.07–2.77] and 1.22 [95%CI 0.78–1.91 for diabetes and BMI > 25 kg/m^2^, respectively) compared to male individuals (HRs 1.36 [95%CI 1.01–1.83] and 1.07 [95%CI 0.75–1.54]). In contrast, the association of hypertension with secondary MACE was higher in males (HR 1.55 [95%CI 1.17–2.06] for hypertension) than in female individuals (HR 1.46 [95%CI 0.91–2.33]) (Table S6). There were no differences observed in associations for cardiac characteristics by gender with no events reported in females for RBBB and second-degree heart block.

Of the three ethnic groups, Indian individuals had the highest prevalence of secondary MACE, 64 (16.7%), followed by 138 (13.9%) events in the Bumiputera individuals, and 29 (12.0%) in the Chinese individuals (Table S7). There was no evidence of effect modification based on ethnic group; however, the statistical power to detect such differences was limited (Supplementary File). The associations for hypertension and LBBB were potentially higher in Bumiputera individuals (HR 1.53 [95%CI 1.08–2.15] for hypertension, and HR 3.58 [95%CI 1.33–9.70] for LBBB), while LVEF ≤ 40% appeared to have a stronger association in Indian individuals (HR 1.67 [95%CI 1.10–2.52]); these findings require further investigation with greater numbers of individuals and events.

## 4. Discussion

In this study, we investigated the incidence and risk factors for secondary MACE. In individuals with a first-documented MI, the incidence of non-fatal secondary MACE was higher than cardiovascular-related mortality by the end of the 1-year follow-up period. Apart from hypertension and diabetes mellitus, other traditional risk factors of MI were not found to be determinants of MACE. NSTEMIs at baseline and conduction disturbances were also associated with a higher risk of MACE. No evidence of differences in risk factors’ effects on MACE was found across age, sex, or ethnic groups, although the statistical power was limited, and certain potentially differing effects warrant further investigation, especially for LBBB and LVEF.

The increased morbidity and mortality of persons with a single MI event were highlighted in several studies [2,19]. The incidence of fatal events reported in this study was generally lower than in local and other reports. The Malaysian NCVD–ACS Registry Report documented an overall incidence of mortality of 17.1% at the 1-year follow-up of index acute coronary syndrome events [20]. The lower incidence of mortality reported in this study may have been a result of several factors: (1) Inclusion of only the first atherosclerotic MI event; (2) differences in baseline characteristics such as younger participants and fewer comorbidities; (3) differences in prognostic factors; and 4) exclusion of unstable angina. Although the mortality rates of the cohort population were lower, the appropriate identification of risk factors affecting prognosis is still essential for reductions in morbidity and mortality.

The incidence of recurrent MI was reported to be between 2.5% and 6.8% in different populations [21,22,23,24]. However, Singaporean patients with their first MI event were reported to have an incidence of recurrent AMI of 6.8% at 1 year [23]. However, there are insufficient reports on recurrent MIs in the Malaysian population. In another Asian population, the 3-year incidence of recurrent AMIs was 5.8%, highlighting that recurrence occurs early after the index MI event [25]. The early occurrence of recurrent MIs was also reported in other studies [21,26]. Diagnosis of STEMI, male gender, older age, and more concurrent comorbidities are the contributing factors of MI recurrence [27,28].

Our findings on hypertension and diabetes as determinants of secondary MACE concur with existing evidence. This increased risk associated with hypertension is the result of several processes that include left ventricular hypertrophy, coronary endothelial dysfunction, abnormal coronary artery remodeling, and coronary microvascular dysfunction [29,30]. During an acute MI event, these different processes give rise to a greater imbalance in oxygen demand and supply, and a worsening of cardiac mechanical dysfunction. Consequently, these factors lead to a larger extent of cardiomyocyte death and other mechanical complications such as cardiogenic shock, cardiac arrest, and possibly heart failure. Although the effects of heart failure require further investigation, their link with hypertension is convincing, as the risk of developing heart failure doubles in those with hypertension compared to those without the presence of hypertension [31]. Several of these processes are also influenced by the state of hyperglycemia [32].

Diabetes mellitus was reported to contribute to more than 40% of MI recurrences [2,33,34]. Its resulting insulin resistance and hyperglycemia cause specific adaptive and maladaptive cellular responses that lead to specific changes in myocardial structure and function [35]. Moreover, the prevalence of atherosclerosis increases with worsening glucose status due to accelerated atherosclerosis and other more direct lipotoxic and glucotoxicity effects [36]. In this study, a high HbA1c was associated with a higher risk of MACE, especially in older (>50 years) individuals. This concurs with evidence reporting the increased risk of macrovascular complications and all-cause mortality with worsening severity of blood glucose impairment [37,38]. We also noted that the prevalence of high HbA1c was higher than the presence of self-reported diabetes at baseline. This raises two concerns: firstly, the high prevalence of undiagnosed diabetes s in these first documented MI individuals, and secondly, the inadequate glycemic control is in those with existing diabetes. With a high prevalence of diabetes in the Malaysian general population [39], this highlights the importance of its early detection prior to the first MI admission, as well as for long-term optimum glycemic control.

We consistently found that diagnoses of NSTEMIs at baseline were a risk factor for secondary MACE. In acute NSTEMIs, the occlusion of coronary vessels does not occur, whereas occlusions of coronary vessels in STEMIs lead to more adverse outcomes; hence, a PCI is recommended to be prioritized in STEMI individuals [40]. Despite the guidelines, there were no large differences in the numbers of PCIs performed between the two types of MIs in this study. However, successful reperfusions are also dependent on other factors, which were not evaluated in this study, such as time to treatment, existing cardiac mechanical dysfunction, and micro- and macrovascular dysfunctions. Thus, further explorations of other determinants of secondary MACE amongst NSTEMI individuals are needed.

In a multi-ethnic population study, individuals without standard modifiable risk factors of CVD demonstrated a higher prevalence of ventricular arrhythmias and higher rates of secondary MACE [41]. The prevalence of ventricular fibrillation, a type of ventricular arrhythmia, was noted to be the highest among all the types of conduction disturbances investigated in this study; however, its association with secondary MACE was not as pronounced as LBBB, RBBB, or even second-degree heart block. Maintaining regular rhythms is essential to both maintaining coronary circulation and cardiac function. In particular, the optimal functioning of the left ventricle ensures sufficient cardiac output to maintain blood flow to other organ systems. Impairment of the left ventricular function would then result in elevated filling pressures and atrial volume overload, and consequently heart failure [42]. Evidence for the association between LBBB and secondary MACE has been well established, resulting in its usage as a diagnostic criterion for new-onset STEMI [14,43,44].

While it is largely possible to prevent the occurrence of an MI, there are notable varia-tions in the risk factors and prevalence of this condition among different ethnicities. De-spite similar environmental conditions, ethnic disparities give rise to diverse risks and outcomes, as exemplified by the South Asian populations in the United Kingdom and the United States, who exhibit a higher susceptibility to CVD compared to other ethnic groups [45,46,47]. Amongst the reported significant contributing factors are diabetes and obesity.

Over the years, there has been a growing body of evidence linking elevated lipopro-tein (a) levels to an increased risk in CHD events, with variations observed among diverse ethnic groups [48,49]. Both elevated lipoprotein (a) levels and the presence of genetic polymorphisms in the LPA gene were found to be independent risk factors of cardiovascular events at 5 years in Chinese individuals [50]. Although significant variations in lipoprotein (a) levels were detected across different ethnicities [51], their impact on the risk of cardiovascular events remained similar [52]. This highlights the importance in taking into consideration the difference in ethnicities and local risk factors for appropriate risk management strategies. 

This study has the advantage of being the first to evaluate the incidence and risk factors of MACE in Malaysians with first-documented MI. Its multi-center cohort design enabled a long-term assessment of outcomes with survival analysis, and a good representation of the Malaysian population from different regions. This study was necessary, as there is limited evidence exploring determinants of secondary MACE in a multi-ethnic population, especially with native ethnic groups. Despite the vast majority of the population being treated in government or state-funded, hospitals, 77% of cardiac catheterization labs are in the private sector [53]. Additionally, coronary care services in Malaysia are focused in urban areas and West Peninsular Malaysia; thus, access to PCI-capable centers may not be timely in certain regions, such as in Borneo. Although numerous studies reported a wide range of risk factors for secondary MACE, this study included variables that are available in both PCI- and non-PCI-capable hospitals.

This study’s measures allow for risk stratification and appropriate treatment strategies. However, given the limited number of Borneo native ethnic groups, these ethnic groups could not be analyzed individually. Thus, studies focusing on these native Borneo ethnic groups are required.

The non-randomized selection of hospitals for inclusion may have led to sampling bias in the included population. Secondary data collection also led to potential inaccuracies in the definitions, diagnosis, and reporting of data, as the data recorded were not recorded for the sole purpose of research. The findings of this study may also not be generalizable to other populations or individuals with recurrent events. Exploration of the study objectives in the otherwise inadequately investigated native ethnic groups of the Southeast Asian region is advantageous, however, the collective analysis of the Bumiputera may undermine the differences between the different native ethnic groups. Since women were underrepresented in this cohort due to the later age of onset [54], there was insufficient power to evaluate gender-based differences in MACE-exposure associations. Additionally, evidence bias of the hazard effect estimates may be present due to the potential omission of balanced covariates, unmeasured confounding, and data censoring. Other potential confounders such as diet, physical inactivity, compliance with medications, extent of cardiac damage, other cardiac-related conditions, and other CVDs such as peripheral vascular disease, were not assessed and may have contributed to the development of secondary MACE. Additional non-biological risk factors, such as political, socioeconomic, and environmental elements, also play a role in determining the quality of life for individuals with a history of MI [55]. Lastly, the imprecision of the effect sizes from the large CIs was attributed to the small sample size.

## 5. Conclusions

As the burden of coronary heart disease is still a challenge, understanding local disease patterns can provide local evidence for a more comprehensive evaluation of prognoses for incorporation into treatment strategies. Secondary prevention through the early diagnosis of traditional risk factors and optimum management of risk factors is essential, especially with the age-declining first-onset MI. Several traditional and cardiac risk factors were associated with a higher risk of secondary major adverse cardiovascular events. In addition to known hypertension and diabetes, we found that the identification of conduction disturbances such as LBBB, RBBB, and AV-blocks in individuals with first-onset MI may be useful for the risk stratification of high-risk individuals.

## Figures and Tables

**Table 1:** Characteristics of cohort individuals at baseline.

Variables	Total Population
***Demography, n* (%)**	
Age, ≤50 years	768 (47.5%)
Male sex	1476 (91.2%)
Ethnicity	
Bumiputera	993 (61.4%)
Chinese	242 (15.0%)
Indian	383 (23.7%)
**Comorbidities, *n* (%)**	
Self-reported hypertension	556 (35.8%)
Self-reported diabetes	448 (28.9%)
Self-reported dyslipidemia	366 (23.6%)
Smoking	942 (61.4%)
Body mass index ≥ 25 kg/m^2^	1119 (78.6%)
**Blood parameters, mean (SD)**	
Total cholesterol, mmol/L	5.7 (1.6)
Low-density lipoprotein, mmol/L	4.0 (1.4)
High-density lipoprotein, mmol/L	1.3 (0.4)
Triglyceride, mmol/L	2.2 (1.1)
Lipoprotein (a), mmol/L	33.6 (28.0)
HbA1c, %	7.1 (2.1)
**Blood parameters, *n* (%)**	
LDL > 2.6 mmol/L	1269 (87.1%)
Lipoprotein (a) > 30 mg/dL	566 (41.4%)
HbA1c > 6.5%	609 (39.9%)
**Cardiac characteristics, *n* (%)**	
NSTEMI	576 (35.6%)
Underwent PCI	979 (60.5%)
STEMI patients with PCI	670 (64.3%)
NSTEMI patients with PCI	309 (53.6%)
*Conduction Disturbances*	
Right-bundle branch block	26 (1.6%)
Left-bundle branch block	18 (1.1%)
First-degree heart block	8 (0.5%)
Second-degree heart block	6 (0.4%)
Complete heart block	20 (1.2%)
Atrial fibrillation	29 (1.8%)
Ventricular fibrillation	45 (2.8%)
Supraventricular tachycardia	42 (2.6%)
Heart failure	79 (6.4%)
LVEF in %, mean (SD)	47.7 (11.3)
LVEF ≤ 40%	316 (26.7%)
LVEF 41–49%	311 (26.3%)
LVEF ≥ 50%	556 (47.0%)
Cardiogenic shock	161 (10.0%)
Cardiac arrest	31 (1.9%)

Abbreviations: LDL—low-density lipoprotein; LVEF—left ventricular ejection fraction; PCI—percutaneous coronary intervention; SBP—systolic blood pressure. Categorical variables are presented as counts (column percentages). Continuous variables are presented as means (standard deviations).

## Data Availability

The datasets generated and/or analyzed for this study are available on reasonable request from the MAVERIK Data Access Committee (maverik@phpc.cam.ac.uk).

## Supplementary Materials

The following supporting information can be downloaded at: https://www.mdpi.com/article/10.3390/jcdd10060250/s1, Table S1: Participating hospitals; Table S2: Characteristics of cohort individuals by age group; Table S3: Characteristics of cohort individuals by ethnicity; Table S4: Associations between risk factors of first MI and risk of MACE; Table S5: Associations between risk factors of first MI and risk of MACE by age; Table S6: Associations between risk factors of first MI and risk of MACE by sex; Table S7: Associations between risk factors of first MI and risk of MACE by ethnicity.
