# Supplementary material for: Risk Factors of Secondary Cardiovascular Events in a Multi-Ethnic Asian Population with Acute Myocardial Infarction: A Retrospective Cohort Study from Malaysia
Source: J Cardiovasc Dev Dis. 2023 Jun 9;10(6):250. doi: 10.3390/jcdd10060250 (PMC10299045; doi:10.3390/jcdd10060250)
Supplement: Supplementary file 1 [file jcdd-10-00250-s001.zip › jcdd-2385035-SI.pdf]

Risk Factors of Secondary Cardiovascular Events in a Multi-ethnic Asian Population with Acute Myocardial Infarction: a Retrospective Cohort Study from Malaysia

1. Supplementary Table S1: Participating hospitals

Region	Included hospitals
Northern region	1. Hospital Sultanah Bahiyah, Kedah<br>2. Hospital Raja Permaisuri Bainun, Perak
East coast region	1. Hospital Raja Perempuan Zainab II, Kelantan
Central region	1. Hospital Kuala Lumpur, Wilayah Persekutuan Kuala Lumpur<br>2. Hospital Serdang, Selangor<br>3. Hospital Shah Alam, Selangor<br>4. Hospital Sungai Buloh, Selangor<br>5. Hospital Tengku Ampuan Rahimah, Selangor
Southern region	1. Hospital Sultanah Aminah, Johor
Borneo	1. Pusat Jantung Sarawak, Sarawak

2. Supplementary Table S2: Baseline characteristics of cohort individuals by age group

Variables	Age ≤50 years		Age >50 years	
	With MACE	No MACE	With MACE	No MACE
	N=101	N=667	N=130	N=720
Demography, n (%)				
Male sex	93 (92)	635 (95)	110 (87)	638 (89)
Ethnicity				
Bumiputera	61 (60)	426 (64)	77 (59)	429 (60)
Chinese	7 (7)	95 (14)	22 (17)	118 (16)
Indian	33 (33)	146 (22)	31 (24)	173 (24)
Comorbidities, n (%)				
Self-reported hypertension	41 (42)	160 (25)	66 (53)	289 (42)
Self-reported diabetes	28 (29)	136 (21)	59 (48)	225 (33)
Self-reported dyslipidaemia	21 (22)	110 (17)	47 (38)	188 (28)
Smoking	72 (74)	450 (70)	54 (44)	366 (54)
Body mass index ≥25 kg/m^2	69 (75)	426 (72)	99 (84)	525 (84)
Blood parameters, mean (SD)				
Total cholesterol, mmol/L	5.5 (1.7)	5.8 (1.5)	5.6 (1.8)	5.6 (1.6)
LDL, mmol/L	4.2 (1.8)	4.2 (1.4)	3.9 (1.7)	3.9 (1.4)
HDL, mmol/L	1.3 (0.5)	1.3 (0.4)	1.3 (0.3)	1.3 (0.4)
Triglyceride, mmol/L	2.1 (1.0)	2.3 (1.2)	2.0 (1.1)	2.1 (1.0)
Lipoprotein (a), mmol/L	37.2 (28.2)	32.8 (28.1)	36.1 (28.1)	33.3 (27.8)
HbA1c, %	7.2 (2.4)	6.0 (2.1)	7.7 (2.3)	7.1 (2.1)
Blood parameters, n (%)				
LDL > 2.6 mmol/L	80 (89)	554 (92)	92 (81)	543 (84)
Lipoprotein (a) > 30mg/dL	44 (51)	226 (40)	50 (46)	246 (41)
HbA1c >6.5%	32 (33)	222 (35)	70 (59)	285 (42)
Cardiac characteristics, n (%)				
NSTEMI	39 (5)	217 (28)	61 (7)	259 (31)
Left bundle branch block	1 (1)	7 (1)	5 (4)	5 (1)
Any arrhythmia	8 (8)	50 (8)	17 (13)	53 (7)
Heart failure	7 (7)	34 (5)	15 (12)	48 (7)
LVEF in %, mean (SD)	43.0 (13.1)	48.9 (10.7)	44.7 (12.7)	47.6 (11.1)
LVEF ≤40%	31 (46)	110 (22)	34 (40)	141 (27)
LVEF 41–49%	14 (21)	141 (28)	22 (26)	134 (25)
LVEF ≥50%	23 (34)	249 (50)	30 (35)	254 (48)
Cardiogenic shock	16 (16)	62 (9)	16 (12)	67 (9)
Cardiac arrest	1 (1)	12 (2)	3 (2)	15 (2)

Abbreviations: HDL – high density lipoprotein; LDL – low density lipoprotein; LVEF – left ventricular ejection fraction; MACE – major adverse cardiovascular events; SBP – systolic blood pressure.
Categorical variables are presented as counts (column percentages). Continuous variables are presented as mean (standard deviation).

3. Supplementary Table S3: Baseline characteristics of cohort individuals by ethnicity

Variables	Bumiputera		Chinese		Indian	
	With MACE	No MACE	With MACE	No MACE	With MACE	No MACE
	N=138	N=855	N=29	N=213	N=64	N=319
Demography						
Age (years), mean (SD)	51.1 (9.2)	49.7 (9.2)	54.5 (6.5)	50.6 (9.3)	50.9 (9.3)	49.8 (10.0)
Age ≤50 years, n (%)	61 (44)	426 (50)	7 (24)	95 (44.6)	33 (52)	173 (54)
Male sex, n (%)	122 (88)	788 (92)	25 (86)	194 (91.1)	56 (88)	291 (91)
Comorbidities, n (%)						
Self-reported hypertension	60 (45)	263 (32)	15 (53)	83 (41.3)	32 (53)	103 (33)
Self-reported diabetes	50 (38)	209 (26)	12 (43)	50 (24.9)	25 (42)	102 (33)
Self-reported dyslipidaemia	42 (32)	184 (22)	12 (43)	45 (22.4)	14 (23)	69 (22)
Smoking	78 (59)	504 (62)	11 (39)	123 (62.8)	37 (62)	189 (61)
BMI ≥25 kg/m^2	101 (80)	585 (77)	21 (81)	133 (76)	46 (79)	233 (83)
Blood parameters, mean (SD)						
Total cholesterol, mmol/L	5.6 (1.9)	5.8 (1.9)	5.5 (2.0)	5.8 (1.5)	5.4 (1.4)	5.6 (1.4)
LDL, mmol/L	4.2 (1.9)	3.9 (1.9)	5.5 (2.0)	4.1 (1.5)	3.8 (1.3)	3.9 (1.2)
HDL, mmol/L	1.2 (0.3)	1.3 (0.3)	1.2 (0.3)	1.3 (0.3)	1.3 (0.6)	1.3 (0.4)
Triglyceride, mmol/L	2.1 (1.1)	2.2 (1.1)	2.0 (1.1)	2.3 (1.4)	2.0 (0.8)	2.2 (1.0)
Lipoprotein (a), mmol/L	35.3 (26.1)	33.8 (29.6)	39.6 (38.8)	30.2 (23.2)	38.0 (27.5)	33.1 (26.2)
HbA1c, %	7.4 (2.3)	7.0 (2.3)	7.7 (2.6)	7.0 (2.1)	7.5 (2.3)	7.1 (2.0)
Blood parameters, n (%)						
LDL > 2.6 mmol/L	105 (85)	676 (88)	18 (72)	170 (86)	49 (89)	251 (87)
Lipoprotein (a) > 30mg/dL	58 (48)	289 (41)	10 (44)	70 (38)	26 (48)	113 (42)
HbA1c >6.5%	60 (46)	298 (37)	15 (58)	82 (39)	27 (47)	127 (42)
Cardiac characteristics, n (%)						
NSTEMI	48 (5)	247 (27)	17 (7)	95 (39)	25 (7)	105 (27)
LBBB	4 (3)	6 (1)	1 (4)	4 (2)	1 (2)	2 (1)
Any arrhythmia	14 (10)	74 (9)	2 (7)	14 (7)	9 (14)	15 (5)
Heart failure	10 (7)	46 (5)	4 (14)	18 (9)	8 (13)	18 (6)
LVEF in %, mean (SD)	44.7 (12.2)	48.4 (12.2)	39.4 (10.5)	46.6 (11.1)	44.8 (14.9)	49.2 (10.4)
LVEF ≤40%	35 (39)	161 (25)	12 (56)	50 (31)	18 (42)	40 (18)
LVEF 41–49%	20 (23)	179 (28)	6 (27)	29 (18)	10 (23)	67 (30)
LVEF ≥50%	34 (38)	303 (47)	4 (18)	82 (51)	15 (35)	118 (52)
Cardiogenic shock	19 (14)	84 (10)	4 (14)	16 (8)	9 (14)	29 (9)
Cardiac arrest	2 (2)	20 (2)	1 (4)	2 (1)	1 (2)	5 (2)

Abbreviations: BMI – body mass index; HDL – high density lipoprotein; LBBB – left bundle branch block; LDL – low density lipoprotein; LVEF – left ventricular ejection fraction; MACE – major adverse cardiovascular events; SBP – systolic blood pressure.

Categorical variables are presented as counts (column percentages). Continuous variables are presented as mean (standard deviation).

4. Supplementary Table S4: Associations between risk factors of first MI and risk of MACE

Variables	Model 1	Model 2	Model 3	Model 4
**Adjusted variables**	None	Age, sex, and ethnicity	Model 2 + hypertension, diabetes, BMI, smoking, Lp (a), and HbA1c	Model 3 + mutual adjustments of cardiac characteristics
**Demographics**				
Age >50 years vs ≤50 years	1.17 (0.90–1.53)	1.14 (0.88–1.49)		
Female	1.51 (1.01–2.24)	1.47 (0.98–2.18)		
Chinese vs Bumiputera	0.86 (0.57–1.28)	0.84 (0.57–1.26)		
Indian vs Bumiputera	1.21 (0.90–1.63)	1.21 (0.90–1.62)		
**Comorbidities**				
Hypertension	1.77 (1.36–2.30)	1.60 (1.22–2.12)		
Diabetes	1.63 (1.23–2.14)	1.46 (1.09–1.97)		
Dyslipidaemia	1.50 (1.11–2.02)	1.41 (0.95–2.08)		
BMI ≥25 kg/m² vs <25 kg/m²	1.04 (0.74–1.45)	1.05 (0.52–2.14)		
Smoking	0.82 (0.63–1.08)	0.93 (0.66–1.30)		
**Blood Parameters**				
LDL >2.6mmol/L vs ≤2.6mmol/L	0.84 (0.26–2.77)	0.88 (0.27–2.83)		
Lp(a) >30mg/dL vs ≤30mg/dL	1.06 (0.60–1.87)	1.05 (0.59–1.86)		
HbA1c >6.5% vs ≤6.5%	1.36 (1.01–1.85)	1.29 (0.94–1.76)		
**Cardiac Characteristics**				
NSTEMI vs STEMI	1.41 (1.08–1.83)	1.37 (1.05–1.79)	1.32 (1.01–1.73)	1.57 (1.07–2.30)
Right bundle branch block	2.37 (1.16–4.81)	2.36 (1.16–4.82)	2.09 (1.02–4.29)	1.88 (0.89–3.95)
Left bundle branch block	2.85 (1.26–6.43)	2.98 (1.32–6.75)	2.86 (1.25–6.55)	1.88 (0.74–4.76)
2nd degree heart block	2.88 (0.71–11.67)	2.70 (0.66–10.99)	2.45 (0.59–10.16)	2.09 (0.48–9.14)
Atrial fibrillation	0.71 (0.23–2.24)	0.67 (0.21–2.11)	0.67 (0.21–2.13)	0.57 (0.17–1.85)
Ventricular fibrillation	1.43 (0.73–2.80)	1.44 (0.73–2.82)	1.51 (0.76–3.00)	1.21 (0.56–2.63)
Supraventricular tachycardia	1.39 (0.69–2.83)	1.49 (0.73–3.04)	1.58 (0.77–3.23)	1.38 (0.64–2.96)
Heart failure	1.59 (1.02–2.47)	1.54 (0.99–2.41)	1.47 (0.94–2.30)	1.18 (0.69–2.01)
LVEF ≤40% vs ≥50%	2.11 (0.33–13.34)	2.19 (0.34–14.21)	2.20 (0.33–14.56)	2.24 (0.27–18.39)
LVEF 41–49% vs ≥50%	1.18 (0.41–3.43)	1.21 (0.41–3.58)	1.19 (0.40–3.53)	1.30 (0.40–4.23)
Cardiogenic shock	1.56 (1.07–2.27)	1.56 (1.07–2.26)	1.56 (1.07–2.27)	1.49 (0.99–2.26)
Cardiac arrest	0.94 (0.35–2.53)	0.93 (0.34–2.50)	0.91 (0.33–2.47)	0.82 (0.29–2.36)

Abbreviations: BMI – body mass index; LDL – low density lipoprotein; Lp(a) – lipoprotein (a); LVEF – left ventricular ejection fraction; MACE – major adverse cardiovascular events.
Values are presented as Hazard Ratio(95% Confidence Interval)
Model 1: Unadjusted Cox regression analysis
Model 2: Cox regression adjusted for age, sex, and ethnicity
Model 3: Cox regression adjusted for Model 2 + hypertension, diabetes, BMI, smoking, lipoprotein (a), and HbA1c
Model 4: Cox regression adjusted for Model 3 + mutually adjusted cardiac characteristics
Dark Background: No comparisons could be made for those coloured as it's the same variables.

5. Supplementary Table S5: Associations between risk factors of first MI and risk of MACE by age

**Risk Factors**	**Age Group**	**Model 2**		**Model 3**	
		**HR (95%CI)**	**p-value**	**HR (95%CI)**	**p-value**
**Comorbidities**					
Hypertension	Age ≤50 years	1.92 (1.29–2.86)			
	Age >50 years	0.90 (0.61–1.33)	0.265		
Diabetes	Age ≤50 years	1.42 (0.93–2.19)			
	Age >50 years	1.15 (0.74–1.77)	0.756		
Dyslipidaemia	Age ≤50 years	1.22 (0.76–1.96)			
	Age >50 years	1.17 (0.71–1.94)	0.800		
BMI ≥25 kg/m^2	Age ≤50 years	1.03 (0.65–1.62)			
	Age >50 years	1.11 (0.83–1.49)	0.625		
Smoking	Age ≤50 years	1.12 (0.72–1.75)			
	Age >50 years	0.93 (0.66–1.32)	0.097		
**Blood Parameters**					
Lp(a) >30mg/dL	Age ≤50 years	1.17 (0.79–1.73)			
	Age >50 years	1.10 (0.76–1.60)	0.778		
HbA1c >6.5%	Age ≤50 years	0.81 (0.53–1.24)			
	Age >50 years	1.57 (1.03–2.38)	0.041		
**Cardiac Characteristics**					
NSTEMI	Age ≤50 years	1.28 (0.86–1.91)		1.23 (0.82–1.83)	
	Age >50 years	1.23 (0.82–1.85)	0.605	1.10 (0.73–1.67)	0.612
RBBB	Age ≤50 years	2.11 (0.52–0.57)		2.15 (0.53–8.81)	
	Age >50 years	1.31 (0.27–6.53	0.843	1.04 (0.21–5.22)	0.966
LBBB	Age ≤50 years	1.00 (0.14–7.17)		1.02 (0.14–7.34)	
	Age >50 years	5.53 (0.64–47.50)	0.093	4.39 (0.50–38.36)	0.127
2nd degree HB	Age ≤50 years	16.17 (2.24–116.73)		18.27 (2.51–133.07)	
	Age >50 years	0.11 (0.01–1.81)	0.136	0.08 (0.01–1.33)	0.108
LVEF ≤40%	Age ≤50 years	1.14 (0.74–1.74)		1.11 (0.72–1.69)	
	Age >50 years	1.03 (0.71–1.50)	0.577	0.96 (0.66–1.40)	0.684
Cardiogenic shock	Age ≤50 years	1.77 (1.04–3.02)		1.74 (1.02–2.98)	
	Age >50 years	0.94 (0.47–1.87)	0.539	0.88 (0.44–1.77	0.656

Abbreviations: BMI – body mass index; HB – heart block; LBBB – left-bundle branch block; Lp(a) – lipoprotein (a); LVEF – left ventricular ejection fraction; MACE – major adverse cardiovascular events; RBBB – right bundle branch block.
Model 2: Cox regression adjusted for sex and ethnicity
Model 3: Cox regression adjusted for Model 2 + hypertension, diabetes, BMI, smoking, HbA1c and lipoprotein (a)
Subgroup analysis was performed using multivariable Cox regression analysis with addition of age-group as interaction term and linear combinations of model parameters
P-value was calculated using Wald test. P-value of >0.05 demonstrates no difference between age ≤50 years and age >50 years.
Dark Background: No comparisons could be made for those coloured as it's the same variables.

6. Supplementary Table S6: Associations between risk factors of first MI and risk of MACE by sex

Risk Factors	Gender	Model 2	
		HR (95%CI)	p-value*
**Comorbidities**			
Hypertension	Female	1.46 (0.91–2.33)	
	Male	1.55 (1.17–2.06)	0.048
Diabetes	Female	1.72 (1.07–2.77)	
	Male	1.36 (1.01–1.83)	0.050
Dyslipidaemia	Female	1.69 (0.95–3.00)	
	Male	1.22 (0.89–1.66)	0.410
Body mass index ≥25 kg/m^2	Female	1.22 (0.78–1.91)	
	Male	1.07 (0.75–1.54)	0.016
Smoking	Female	1.66 (0.41–6.71)	
	Male	0.85 (0.64–1.13)	0.764
**Blood Parameters**			
Lipoprotein (a) >30mg/dL	Female	1.10 (0.61–2.01)	
	Male	1.19 (0.91–1.57)	0.184
HbA1c >6.5%	Female	1.45 (0.89–2.36)	
	Male	1.12 (0.84–1.48)	0.884
**Cardiac Characteristics**			
NSTEMI	Female	1.22 (0.71–2.09)	
	Male	1.43 (1.08–1.89)	0.511
Left bundle branch block	Female	2.32 (0.27–20.06)	
	Male	2.81 (1.15–6.84)	0.688
LVEF ≤40%	Female	1.64 (0.93–2.87)	
	Male	1.09 (0.80–1.47)	0.207
Cardiogenic shock	Female	1.75 (0.61–5.01)	
	Male	1.52 (1.02–2.27)	0.739

Abbreviations: LVEF – left ventricular ejection fraction; MACE – major adverse cardiovascular events.
Model 2: Cox regression adjusted for age, and ethnicity
Subgroup analysis was performed using multivariable Cox regression analysis with addition of gender as interaction term and linear combinations of model parameters
P-value was calculated using Wald test. P-value of >0.05 demonstrates no difference between male and female groups.

7. Supplementary Table S7: Associations between risk factors of first MI and risk of MACE by ethnicity

Risk Factors	Ethnic Group	Model 2	
		HR (95%CI)	p-value*
**Comorbidities**			
Hypertension	Bumiputera	1.53 (1.08–2.15)	0.697
	Chinese	0.79 (0.45–1.39)	
	Indian	1.35 (0.88–2.06)	
Diabetes	Bumiputera	1.47 (1.03–2.09)	0.916
	Chinese	0.94 (0.50–1.77)	
	Indian	1.18 (0.75–1.86)	
Dyslipidaemia	Bumiputera	1.31 (0.91–1.89)	0.282
	Chinese	1.01 (0.58–2.00)	
	Indian	0.95 (0.53–1.71	
Body mass index ≥25 kg/m^2	Bumiputera	1.01 (0.66–1.56)	0.706
	Chinese	0.86 (0.55–1.33)	
	Indian	1.15 (0.82–1.59)	
Smoking	Bumiputera	0.89 (0.63–1.27)	0.109
	Chinese	0.57 (0.30–1.07)	
	Indian	1.30 (0.89–1.91)	
**Blood Parameters**			
Lipoprotein (a) >30mg/dL	Bumiputera	1.09 (0.78–1.53)	0.969
	Chinese	0.86 (0.49–1.53)	
	Indian	1.28 (0.82–2.00)	
HbA1c >6.5%	Bumiputera	1.18 (0.84–1.67)	0.457
	Chinese	0.95 (0.54–1.67)	
	Indian	1.02 (0.65–1.60)	
**Cardiac Characteristics**			
NSTEMI	Bumiputera	1.42 (1.01–2.00)	0.762
	Chinese	0.86 (0.50–1.48)	
	Indian	1.09 (0.68–1.74)	
Left bundle branch block	Bumiputera	3.58 (1.33–9.70)	0.849
	Chinese	0.47 (0.05–4.19)	
	Indian	0.89 (0.10–7.97)	
LVEF ≤40%	Bumiputera	0.81 (0.56–1.16)	0.052
	Chinese	0.95 (0.54–1.68)	
	Indian	1.67 (1.10–2.52)	
Cardiogenic shock	Bumiputera	1.50 (0.92–2.43)	0.917
	Chinese	1.06 (0.36–3.14)	
	Indian	1.25 (0.56–2.77)	

Abbreviations: LVEF – left ventricular ejection fraction; MACE – major adverse cardiovascular events.
Model 2: Cox regression adjusted for age and sex

Subgroup analysis was performed using multivariable Cox regression analysis with addition of ethnic group as interaction term and linear combinations of model parameters
P-value was calculated using Wald test. P-value of >0.05 demonstrates no difference between Bumiputera and other ethnic groups.
